# Consumption of low-moderate level arsenic contaminated water does not increase spontaneous pregnancy loss: a case control study

**DOI:** 10.1186/1476-069X-13-81

**Published:** 2014-10-13

**Authors:** Michael S Bloom, Iulia A Neamtiu, Simona Surdu, Cristian Pop, Ioana Rodica Lupsa, Doru Anastasiu, Edward F Fitzgerald, Eugen S Gurzau

**Affiliations:** Department of Environmental Health Sciences, University at Albany, State University of New York, School of Public Health, Rensselaer, NY USA; Department of Epidemiology and Biostatistics, University at Albany, State University of New York, School of Public Health, Rensselaer, NY USA; Environmental Health Center, Cluj-Napoca, Romania; University of Medicine and Pharmacy “Victor Babes”, Timisoara, Romania; Obstetrics and Gynecology Department of the Emergency County Hospital, Timisoara, Romania; University of Medicine and Pharmacy “Iuliu Haţieganu”, Cluj-Napoca, Romania

**Keywords:** Arsenic, Cigarette smoking, Drinking water, Human reproduction, Pregnancy loss, Spontaneous abortion

## Abstract

**Background:**

Previous work suggests an increased risk for spontaneous pregnancy loss linked to high levels of inorganic arsenic (iAs) in drinking water sources (>10 μg/L). However, there has been little focus to date on the impact of low-moderate levels of iAs in drinking water (<10 μg/L). To address this data gap we conducted a hospital-based case–control study in Timis County, Romania.

**Methods:**

We recruited women with incident spontaneous pregnancy loss of 5–20 weeks completed gestation as cases (n = 150), and women with ongoing pregnancies matched by gestational age (±1 week) as controls (n = 150). Participants completed a physician-administered questionnaire and we collected water samples from residential drinking sources. We reconstructed residential drinking water exposure histories using questionnaire data weighted by iAs determined using hydride generation-atomic absorption spectrometry (HG-AAS). Logistic regression models were used to generate odds ratios (OR) and 95% confidence intervals (CI) for associations between iAs exposure and loss, conditioned on gestational age and adjusted for maternal age, cigarette smoking, education and prenatal vitamin use. We explored potential interactions in a second set of models.

**Results:**

Drinking water arsenic concentrations ranged from 0.0 to 175.1 μg/L, with median 0.4 μg/L and 90^th^%tile 9.4 μg/L. There were no statistically significant associations between loss and average or peak drinking water iAs concentrations (OR 0.98, 95% CI 0.96-1.01), or for daily iAs intake (OR 1.00, 95% CI 0.98-1.02). We detected modest evidence for an interaction between average iAs concentration and cigarette smoking during pregnancy (P = 0.057) and for daily iAs exposure and prenatal vitamin use (P = 0.085).

**Conclusions:**

These results suggest no increased risk for spontaneous pregnancy loss in association with low to moderate level drinking water iAs exposure. Though imprecise, our data also raise the possibility for increased risk among cigarette smokers. Given the low exposures overall, these data should reassure pregnant women and policy makers with regard to the potential effect of drinking water iAs on early pregnancy, though a larger more definitive study to investigate the potential risk increase in conjunction with cigarette smoking is merited.

## Background

The consumption of contaminated groundwater is the primary source of human exposure to inorganic arsenic (iAs), a highly toxic metalloid present naturally in the earth’s crust [1]. Worldwide, reports describe contamination of drinking water sources ranging from less than 0.5 μg/L to more than 5,000 μg/L [2, 3]. Adverse human health effects, including increased rates of spontaneous clinical pregnancy loss have been reported in so-called ‘arsenic endemic’ areas, regions where drinking water iAs contamination exceeds 10 μg/L and frequently 50 μg/L [4, 5]. The results of epidemiologic studies conducted among pregnant women residing in the iAs endemic regions of Hungary [6], Chile [7], Bangladesh [8–15], India [16–18] and Mongolia [19], demonstrate increases in the risk of loss in association with drinking water iAs exposure >10 μg/L.

Despite the evidence from arsenic-endemic regions there are very few data available to characterize the dose–response relation between low-moderate drinking water iAs contamination and adverse human health effects [20]. Little information is available to assess risks in the low-moderate range, that is, concentrations below 10 μg/L, to which a large population is exposed globally [2, 21]. Only a single epidemiologic study has been reported in which pregnancy loss was assessed in association with low-moderate drinking water iAs exposures; a 50% increase in odds for pregnancy loss was reported for concentrations as low as 0.8-1.3 μg/L, albeit not of statistical significance [22]. Given the widespread nature of low-moderate drinking water iAs contamination and a 12-14% baseline incidence of spontaneous clinical pregnancy loss in humans [23, 24], even a modest increase in risk will attribute a large number of events and thus comprise a substantial public health burden. There is a critical need for epidemiologic studies to assess and quantify the risk and to help guide prevention efforts. As an initial step to help address this gap we conducted a pilot investigation in Timis County, Romania, an area known for low-moderate iAs contamination of drinking groundwater sources [25].

## Methods

### Study sample

We enrolled 150 incident cases of clinically-recognized spontaneous pregnancy loss prior to 20 weeks completed gestation as case participants, and 150 singleton pregnancies receiving prenatal care services as control participants, between December 2011 and January 2013. Prenatal care is provided at no cost and is compulsory in Romania, as is treatment for spontaneous loss. All new pregnancies are registered by family physicians, and referred to an obstetrician for evaluation and clinical examination within one month of gestation. Follow-up prenatal care visits are required in the 2^nd^ and 3^rd^ trimesters. Based on clinical experience, we estimate that 85% of pregnant women comply with the policy and receive pregnancy services; more than 99% of women do so at no cost using county medical facilities. The majority of Timis County pregnancies are attended at the Obstetrics and Gynecology Department of the Emergency County Hospital (Bega Hospital), a comprehensive 1174 bed government medical facility located in the county capital city Timisoara and the site for our study. Women receiving prenatal care for ongoing pregnancies of similar duration to each case-participant (±1 week) were enrolled as controls. A nurse explained the study to women upon admission, obtained consent and completed a basic medical intake form as part of routine clinical evaluation. Participants were remunerated by $20.00 U.S. equivalent in Romanian Lei. The study protocol was approved by the Institutional Review Boards of the County Emergency Hospital in Timisoara and the University at Albany, State University of New York. All participants provided informed consent prior to study enrollment.

After admission, participants completed a physician-administered questionnaire concerning demographics, socioeconomic and lifestyle factors, as well as medical, gynecologic, and occupational histories that were validated during completion of the New York State Angler Cohort Study Prospective Pregnancy Study [26]. Additional questions regarding residential and workplace drinking water consumption history were validated during completion of the Arsenic Health Risk and Molecular Epidemiology (ASHRAM) study [27] We abstracted clinical data from basic medical intake forms completed by nursing staff, such as personal and family medical history, reproductive history and menstrual characteristics. Urine specimens, for a future speciated arsenic analysis, and blood specimens for future genetic and nutritional analyses were collected at the time of the interview, and archived at -20 C.

### Water analysis

Trained study staff collected drinking water samples on-site into 50 mL screw-top polyethylene containers previously decontaminated with nitric acid (HNO_3_) and rinsed. Containers were twice partly filled with sample water (~33 mL), tightly capped, shaken vigorously and emptied prior to collection to the upper edge of the container. We immediately added 100 μL of concentrated analytical grade HNO_3_ as a preservative_._ Water samples were stored in a cooler with ice until delivery to the Environmental Health Center laboratory (Cluj-Napoca, Romania) for analysis. Sample water (25 mL) was mixed with 10 mL HCL and 2 mL aqueous 5% KI and 5% ascorbic acid (m/m). Samples were gently heated to 50°C for 15 minutes to reduce As^5+^ to As^3+^ and then cooled at room temperature. We transferred the sample to a volumetric flask with 50 mL nominal capacity and diluted to volume with de-ionized water. Hydrides were generated using 0.3% NaBH_4_ dissolved in 0.1% NaOH and 3% HCL (m/v), transported to a quartz cell heated to 960°C and analysed by a Zeenit 700p atomic absorption spectrometer (Analytikjena, Jena, Germany). We purchased reagents from Merck (Darmstadt, Germany), Sigma-Aldrich (Steinheim, Germany) and Chem Lab (Zedelgem, Belgium).

Arsenic calibration standard concentrations were 0.0, 1.0, 3.0, 7.0 and 10.0 μg/L. Quality control (QC) was maintained using a second set of source calibration solutions prepared identically to the calibration standards. Measured values of prepared QC samples were within two standard deviations of the calibration standards (e.g., for the 5 μg/L QC sample a value between 4.67 μg/L and 5.33 μg/L was acceptable, given the standard deviation of 0.166). The method detection limit (MDL) was 0.5 μg/L. For the purposes of reporting concentrations negative values were censored as zero, although no values were censored below the MDL during regression analysis to avoid the introduction of bias this may cause [28].

### Exposure indices

Exposure was defined by three indices: 1) average iAs concentration (μg/L), comprising the mean levels measured in up to two residential drinking water sources; 2) peak iAs concentration (μg/L), the highest iAs level measured in up to two residential drinking water sources; and 3) daily exposure (μg/day), the product of average reported daily residential drinking water consumption from non-bottled sources (including teas, coffees, other water-mixed beverages and soups) during pregnancy multiplied by the average iAs concentration.

### Statistical analysis

We examined distributions and evaluated bivariate associations among pregnancy loss, exposure indices and potential confounding factors. We generated logistic regression models, conditioned on gestational age, using iAs exposure indices as predictors, pregnancy loss as the outcome, and adjusted for confounders selected using literature evidence coupled to directed acyclic graphs (DAGs). DAGs employ causal graphing theory to allow identification of minimally sufficient covariates sets to control for confounding while minimizing bias [29]. We included age [30, 31] and smoking as continuous variables [32, 33], categorical education (<high school, high school, university) as a proxy for socioeconomic status [34–36], and self-reported use of perinatal vitamin supplements as a dichotomous variable [37, 38]. The impact of body mass index (BMI), a continuous proxy for overall nutritional status, was assessed and defined as the weight divided by the square of height measured at the time of the study interview. We operationalized exposure indices as continuous predictors to maximize statistical power, and as tertiles of the control distribution to assess non-linear associations.

We also evaluated departures from multiplicativity by incorporating product terms between continuous exposures, expressed as a 10 units increase, and covariates *a priori* specified as likely to modify iAs-pregnancy loss associations into additional regression models. These included age and BMI (pre-pregnancy and measured at the time of the interview) dichotomized at the median of the control distribution, urban vs. rural residence, education, smoking during pregnancy (no/yes), use of prenatal vitamins, and self-reported reproductive history, including previous pregnancy, live birth, and pregnancy loss (no/yes). We further scrutinized product terms significant by Wald test, and characterized the individual effects and joint effects relative to no exposure where these departed from multiplicativity [39].

For all models, we excluded influential observations, described as DfBeta ≥ |1.0| for either exposure or product terms, and repeated the analysis [40]. Under our incidence density case sampling strategy exponentiation of conditional logistic regression model coefficients and their corresponding 95% confidence intervals (95% CI) provided unbiased odds ratio (OR) estimates of the underlying population incidence rate ratio [41]. SAS v.9.3 (SAS Institute Inc., Cary, NC) was used for data analysis and statistical significance was defined as P < 0.05 for main effects and P < 0.10 for product terms. In keeping with the preliminary nature of our study, no adjustment was made for type-1 error inflation due to multiple-comparisons.

## Results

### Univariate and bivariate analyses

One-hundred seventy spontaneous pregnancy losses occurred at Bega Hospital over the study period, 88% of women with losses consented to participate in our study as cases; 83% of women approached with ongoing pregnancies agreed to participate as controls. As described by Table 1, cases and controls were approximately 8.0 and 8.5 weeks pregnant on average, respectively, with a range of 5.0-20.0 weeks. Cases were significantly older than controls and less likely to have reported use of prenatal vitamins. Pre-pregnancy BMI and BMI measured at the time of the interview were similar for cases and controls, although we detected modest increases between pre-pregnancy report and measured value at the time of the interview (δ = 0.31 kg, P = 0.02 and δ = 0.45 kg, P < 0.0001, respectively). Cases and controls were similar in terms of urban vs. rural residence, marital status and formal education. No differences were detected with respect to cigarette smoking and alcohol consumption during pregnancy. Cases reported significantly more prior pregnancies and live births than controls, although the number of prior losses (including elective terminations) was similar.

**Table 1:** **Study sample characteristics by case–control status**

Factor	Cases (n = 150)			Controls (n = 150)			P-value ^a^
	n	Median, (%)	Range	n	Median, (%)	Range	
Demographic:							
Gestational age (weeks)	150	8.0	5-20	150	8.5	5-20	0.256
Maternal age (years)	150	30.0	18.0-42.3	150	27.6	18.8-41.9	0.029
BMI, pre-pregnancy (kg/m^2^)	148	21.6	15.9-46.6	148	21.8	15.1-36.7	0.838
BMI, interview (kg/m^2^)	148	22.1	15.9-46.6	150	22.2	16.2-38.5	0.702
Urban residence	108	(72.0)	-	98	(65.3)	-	0.258
Married, living as married	129	(86.0)	-	120	(80.0)	-	0.160
Education^b^							
University diploma	60	(40.3)	-	68	(45.3)	-	0.652
High school diploma	51	(34.2)	-	45	(30.0)	-	-
No high school diploma	38	(25.5)	-	37	(24.7)	-	-
Health-related behaviors:							
Prenatal vitamins during pregnancy	67	(44.7)	-	97	(64.7)	-	0.0005
Smoked during pregnancy	41	(27.3)	-	36	(24.0)	-	0.515
Average daily cigarettes^c^	41	10	2-50	36	10	1-20	0.267
Alcohol during pregnancy	12	(8.0)	-	13	(8.7)	-	0.842
Work stress during pregnancy	63	(42.0)	-	71	(47.3)	-	0.339
Home stress during pregnancy	62	(41.3)	-	57	(38.0)	-	0.522
Reproductive history:							
Prior pregnancies	150	1	0-14	150	1	0-9	0.038
Prior live births	150	0	0-12	150	0	0-3	0.021
Prior losses	150	0	0-7	150	0	0-8	0.378

^a^Wilcoxon signed-rank test or McNemar’s *χ* ^2^-test for difference between cases & controls; ^b^n = 1 missing case value; ^c^Among n = 77 smokers.

Distributions for iAs exposure metrics are described in Table 2. Although the range of water concentrations was large overall (0–175.10 μg/L iAs), more than 90% of participants used water sources contaminated by less than 9.39 μg/L iAs, with a median average concentration of 0.41 μg/L. Five participants (n = 2 cases, n = 3 controls) were exposed to residential water sources with iAs concentration in excess of 50 μg/L on average. In general, unadjusted concentrations were higher among controls than among cases, though differences were not significant. Cases reported significantly higher water consumption from residential sources than controls (δ = 0.3 L, P = 0.002). Most women (n = 299) had resided in the study residence for at least one year (median = 5.5, range 0.5-42).

**Table 2:** **Distribution of inorganic arsenic (iAs) exposure indices by case–control status**

Factor	Cases (n = 150)			Controls (n = 150)			P-value ^a^
	Mean	Median	Range	Mean	Median	Range	
Daily water consumption (L)^b^	1.49	1.50	0.5-5.0	1.26	1.20	0.0-4.0	0.002
Average iAs (μg/L)^c^	4.06	0.17	0-175.10	4.32	1.28	0-130.30	0.169
Peak iAs (μg/L)^d^	4.64	0.17	0-175.10	4.99	2.27	0-130.30	0.121
Daily iAs (μg/day)^e^	7.10	0.24	0-350.20	5.16	1.45	0-83.15	0.130

^a^Wilcoxon signed-rank test; ^b^average water consumed from non-bottled sources, including direct consumption, teas, coffees, mixed beverages and soups; ^c^mean iAs level measured in up to two residential drinking water sources; ^d^the highest iAs concentration measured in up to two residential drinking water sources; ^e^product of ‘daily water consumption’ multiplied by ‘average iAs.’

### Multivariable analyses

We generated logistic regression models for each of three drinking water iAs indices as predictors, conditioned on gestational age, and adjusted for maternal age, cigarette smoking during pregnancy, education and prenatal vitamin use (Table 3). Data for two participants were excluded from the analysis due to influential observations. Effect estimates, expressed as ORs for a one unit increase in exposure, were close to 1.0 for arsenic exposure in all models, maternal age was consistently and significantly associated with increased odds for pregnancy loss, and prenatal vitamin use was consistently and significantly associated with decreased odds for loss. Results were similar when expressing exposure by tertiles, when excluding participants exposed to >50 μg/L iAs on average and when adjusted for BMI (data not shown).

**Table 3:** **Covariate-adjusted odds ratios for spontaneous pregnancy loss in association with continuous iAs exposure (n = 300)**

Predictor	OR ^a^	95% CI
Average iAs (μg/L)^b,c^	0.98	0.96, 1.01
Age	1.05	1.00, 1.10
Smoking	1.02	0.98, 1.06
High School	1.24	0.62, 2.47
University	0.94	0.47, 1.91
Prenatal vitamins	0.43	0.25, 0.74
Peak iAs (μg/L)^c,d^	0.98	0.96, 1.01
Age	1.05	1.00, 1.10
Smoking	1.02	0.98, 1.06
High School	1.23	0.62, 2.46
University	0.96	0.47, 1.93
Prenatal vitamins	0.43	0.25, 0.74
Daily iAs (μg/day)^c,e^	1.00	0.98, 1.02
Age	1.05	1.01, 1.10
Smoking	1.02	0.98, 1.06
High School	1.25	0.63, 2.49
University	1.00	0.49, 2.03
Prenatal vitamins	0.44	0.25, 0.75

^a^Logistic regression models conditioned on gestational age (weeks) and adjusted for maternal age (years), cigarette smoking during pregnancy (average daily cigarettes), education (<High School, High School, University) and prenatal vitamin use (no/yes); ^b^mean iAs level measured in up to two residential drinking water sources; ^c^n = 2 influential observations excluded; ^d^the highest iAs level measured in up to two residential drinking water sources; ^e^product of ‘daily water consumption’ multiplied by ‘average iAs.’

iAs, inorganic arsenic; CI, confidence interval; OR, odds ratio.

In interaction models, the association between iAs exposure and pregnancy loss was modified by cigarette smoking during pregnancy (Table 4). A 10 μg/L increase in average drinking water iAs corresponded to increased odds for loss in smokers, but a possible decrease among non-smokers. We generated analogous results using peak drinking water iAs or daily iAs as the exposure, although the latter was modest. The association between daily iAs exposure and loss was modified by prenatal vitamin use, in which a 10 μg increase in daily iAs exposure corresponded to decreased odds for loss in users with no effect among non-users (Table 4). We did not detect interactions with average or peak drinking water iAs concentrations and prenatal vitamin use. Comparable results were produced by excluding participants exposed to >50 μg/L iAs on average (data not shown). We found no evidence for effect modification by age, BMI, urban vs. rural residence, marital status, education or reproductive history (data not shown).

**Table 4:** **Covariate-adjusted odds ratios for spontaneous pregnancy loss in association with continuous iAs exposure, by cigarette smoking (n = 300)**

Product-term entered	Risk/Protector factor and iAs exposure	Cigarette smoking ^a^		Prenatal vitamin use ^b^	
		OR ^c^	95% CI	OR ^c^	95% CI
Average iAs (μg/L)^d^ × Risk/Protector factor	No				
	0.0 μg/L iAs	1.00	Reference	1.00	Reference
	10.0 μg/L iAs	0.70	0.44, 1.09	1.12	0.72, 1.72
	Yes				
	0.0 μg/L iAs	0.97	0.51, 1.83	0.50	0.27, 0.90
	10.0 μg/L iAs	1.75	0.75, 4.10	0.30	0.13, 0.67
	P-value for interaction	0.057		0.159	
Peak iAs (μg/L)^e^ × Risk/Protector factor	No				
	0.0 μg/L iAs	1.00	Reference	1.00	Reference
	10.0 μg/L iAs	0.73	0.52, 1.04	1.07	0.71, 1.63
	Yes				
	0.0 μg/L iAs	0.97	0.51, 1.82	0.47	0.26, 0.86
	10.0 μg/L iAs	1.69	0.76, 3.74	0.35	0.18, 0.70
	P-value for interaction	0.053		0.298	
Daily iAs (μg/day)^f^ × Risk/Protector factor	No				
	0.0 μg/day iAs	1.00	Reference	1.00	Reference
	10.0 μg/day iAs	0.78	0.58, 1.06	1.04	0.80, 1.34
	Yes				
	0.0 μg/day iAs	0.94	0.49, 1.78	0.54	0.30, 0.98
	10.0 μg/day iAs	1.41	0.73, 2.74	0.31	0.15, 0.63
	P-value for interaction	0.048		0.085	

^a^n=2 influential observations excluded;

^b^n=4 influential observations excluded;

^c^Logistic regression models incorporating continuous exposure expressed as a 10 units increase, conditioned on gestational age (weeks), and including product-term, adjusted for maternal age (years), cigarette smoking during pregnancy (average daily cigarettes), education (<High School, High School, University) and prenatal vitamin use (no/yes); ^d^mean iAs level measured in up to two residential drinking water sources; ^e^the highest iAs level measured in up to two residential drinking water sources; ^f^product of ‘daily water consumption’ multiplied by ‘average iAs.’

iAs, inorganic arsenic; CI, confidence interval; OR, odds ratio.

## Discussion

In this hospital-based case–control study we report no statistically significant association between low-moderate level drinking water iAs contamination and spontaneous clinical pregnancy loss. After adjustment for confounders, effect estimates were close to the null for average and peak drinking water iAs concentrations, as well as for daily drinking water iAs exposure. Assessing interactions, we detected modest evidence for increased odds among cigarette smokers, as well as for decreased odds among prenatal vitamins users.

The detection of age as a risk factor and use of prenatal vitamins as a protective factor in our multivariable models are similar to the results reported by previous studies. Each additional maternal year increased the odds for pregnancy loss by approximately 1.05-1.06-fold, consistent with prior reports of increased risks among women in their early 20s to early 40s [31], and likely associated at least in part with increased rates of chromosomal aneuploidy [42]. Use of prenatal vitamins was associated with a 0.4-0.5-fold decrease in the odds for pregnancy loss, similar to the effects sizes previously reported for self-reported vitamin supplement use in early pregnancy [38]. A review of supplementation trials did not support a protective role, and thus self-reported prenatal vitamin use may reflect other protective health-related behaviors, rather than an effect of vitamins *per se* [43]. In contrast, we did not detect a previously reported odds increase in association with cigarette smoking [44]. Approximately 26.9% of Romanian women aged 15–45 years smoked a daily average of 14.1 cigarettes in 2011 [45], similar to our controls. Thus, the result may be a chance occurrence. A larger sample will allow for a definitive explanation.

Most previous investigations of drinking water exposure and spontaneous pregnancy loss were conducted in populations employing highly contaminated sources, generally >50 μg/L iAs [5]. Two large ecologic studies reported increased rates of pregnancy loss among women residing in regions of Hungary [6] and Chile [7] with high level groundwater iAs contamination. A third reported a 1.16-fold (95% CI 0.001-1.34) increased risk for spontaneous abortion in Hungarian women using drinking water contaminated by >20 μg/L compared to <10 μg/L iAs [46]. Several small cross-sectional studies (n = 16 to n = 192) conducted in Bangladesh and India [8–11, 16] reported loss rates 1.6-fold to 3.3-fold higher for women employing drinking water sources contaminated by 50–1,474 μg/L iAs compared to 0–400 μg/L iAs. In larger cross-sectional studies, a 1.75-fold increase in the rate of loss (P < 0.05) was reported for 300 Bengali women exposed to 10–600 μg/L iAs [18] and a 2.7-fold increase in the rate of loss (95% CI 0.8–8.40) was reported for 323 Mongolian women exposed to >50 μg/L iAs [19]. Similar pregnancy loss rates were reported for women exposed to >200 μg/L iAs compared to <50 μg/L iAs in a cross-sectional study of 587 West Bengal pregnancies [17]. A prospective study of 29,134 Bangladeshi women reported a 1.14-fold increased risk (95% CI 1.04-1.25) for all spontaneous and induced pregnancy losses among women exposed to >50 μg/L iAs [13]. The same group of investigators more recently reported a 1.4-fold risk increase (95% CI 0.96-2.2) for spontaneous loss before 28 weeks completed gestation in the highest compared to the lowest quintiles of urine total iAs, yet without evidence for a dose–response relation [15].

To our knowledge, only one previously published study assessed spontaneous pregnancy loss in a population exposed primarily to drinking water sources contaminated by <10 μg/L iAs. In that 1976–1978 hospital-based U.S. case–control study [22], increased odds for spontaneous loss was reported in association with exposure to municipal drinking water contaminated by 0.8-1.3 μg/L iAs (OR 1.2, 95% CI 1.0-1.6), or 1.4-1.9 μg/L iAs (OR 1.7 95% CI 0.7-4.2) compared to <0.8 μg/L iAs. The use of routinely water quality data reports was likely to have introduced non-differential exposure measurement misclassification, and so the results may have been biased towards the null hypothesis. Our work expands upon this only study of low-level drinking water exposures, as half of our participants were exposed to an average of less than 0.41 μg/L iAs and most were exposed to less than 9.39 μg/L iAs on average. However, point estimates were fairly precise and close to the null hypothesis, indicating no likely increased risk.

The odds increase for pregnancy loss in association with drinking water iAs exposure was limited to cigarette smokers during pregnancy in this study, whereas there was a suggested protective iAs effect among non-smokers. However subgroup numbers were small, the effect estimates imprecise and the latter unexpected association contradicted our initial hypothesis. Furthermore, ours is the first report of iAs associated adverse pregnancy effects among only smokers and so may reflect a chance occurrence. Still, an arsenic associated increased bladder cancer risk was also reported for smokers only in an American study [47]. Arsenic induced increases in the levels of reactive oxygen species (ROS) have been documented by experimental [48] and observational studies [49]. Excess ROS produces oxidative stress (OS), inducing lipid peroxidation, oxidizing proteins and nucleic acids, and leading to abnormal function and DNA lesions [50]. Glutathione serves a critical role in moderating OS [51] and is also an important factor for iAs metabolism [52]. Observational evidence indicates reduced levels of circulating glutathione among women with long-term exposure to iAs >10 μg/L [53], and similar observations have been made for cigarette smokers [54]. Cigarette smokers might be more susceptible than non-smokers to toxic effects from low-dose arsenic exposure. In contrast, there was a suggestion for a possible protective effect for iAs exposure among women using prenatal vitamins, although the interaction was of borderline statistical significance, and was not detected using average or peak water iAs concentrations. Thus, this result may also reflect a chance occurrence.

Concentrations of iAs measured among sources used by study participants were lower than we had anticipated *a priori* based on levels previously reported by Timis County authorities. A median of 4.96 μg/L iAs (range 0.01-71.66) was reported for 96 western Timis County drinking water sources sampled during 2005–2006 (Unpublished data). While these levels were greater than for the current study on average, it is important to note that only groundwater sources (i.e., wells) were captured in the Timis County Health Department survey and our current study collected samples from drinking water sources supplied by both surface and groundwater. The limited variability of exposures likely resulted from usage of municipal surface water sources low in iAs, with overall concentrations possibly below the threshold for an effect. In fact, when limited to only groundwater well samples, we measured a median of 3.61 μg/L iAs (range 0.00-175.10) in 119 participants. Significant loss increases have been reported only with >20 μg/L iAs [46]. Urine iAs biomarkers will be necessary to fully assess the impact. Our study population comprised clinically recognized pregnancies which also may have shifted the exposures towards lower values if iAs reduced pregnancy, a hypothesis we intend to explore in a separate manuscript.

Several issues limit the results of our pilot study. We captured only clinically-recognized pregnancy losses, which might have introduced a competing-risk bias towards the null hypothesis should pre-clinical loss be associated with iAs exposure [55]; thus, true effects may be even greater than we report here. A large number of pregnancy losses are induced in our study population; 71% of the abortions treated in Timis County (i.e., Bega Hospital) were elective during 2008 [56]. Elective terminations were excluded from our study, yet we do not suspect differential exposure between women with induced and spontaneous losses and so bias is unlikely. We also did not accommodate exposure to other potential causes of pregnancy loss that may ‘track’ with iAs. Variable exceedances of European Union (EU) air quality standards for CO and NO_x_ occurred within limited areas of Timisoara during 2008 [57]. However, the evidence for associations with spontaneous loss is weak, and purported associations are low [58]. Furthermore, we identified no associations using ‘urban residence’ as a proxy for air pollution exposure. We also did not incorporate assessment of iAs metabolism, which may have introduced a bias towards the null hypothesis. Wide between-person variability is reported for iAs detoxification, governed by genetic polymorphisms [59] and nutritional factors [52]. Likewise, our use of self-reported health-behaviors data including cigarette smoking, alcohol consumption and prenatal vitamin use may have introduced misclassification, but the nature of any misreport is difficult to predict. Finally, reproduction is a couple-level function [60] although we captured only maternal data. We believe maternal exposures are likely to be of greater importance, but evidence also suggests the relevance of male factors to pregnancy loss [61, 62].

## Conclusions

In conclusion, our study results indicate no increased risk for spontaneous pregnancy loss of <20 weeks completed gestation among women with low to moderate drinking water iAs exposure. Prenatal vitamin use appeared to be protective. While our results are internally valid, we recommend caution in generalizing to other populations and to groups with dissimilar levels of iAs exposure, dietary habits and health-related behaviors. Given the low overall exposures our study should reassure mothers, clinicians and policy makers in Timis County. Still, our data raise the possibility for an increased risk among women smoking cigarettes during pregnancy, which merits a more definitive investigation. These results add to our knowledge as to how low-levels of iAs impact human reproduction.

## Abbreviations

iAs: Inorganic arsenic
BMI: Body mass index
CI: Confidence interval
δ: Difference
OR: Odds ratio
OS: Oxidative stress
ROS: Reactive oxygen species.
